# Mapping the Landscape of Medical AI Research in Korea Using Topic Modeling

**DOI:** 10.3390/healthcare14040549

**Published:** 2026-02-23

**Authors:** Heejang Yun, Yoonhee Lee

**Affiliations:** 1 Department of Nursing, Bucheon University, 56 Sosa-ro, Bucheon-si 14774, Republic of Korea; nursing@bc.ac.kr; 2 Department of Nursing, Woosong College, 171 Dongdaejeon-ro, Dong-gu, Daejeon 34606, Republic of Korea

**Keywords:** artificial intelligence, medical AI, topic modeling, keyword network analysis, research trends

## Abstract

**Background**/**Objectives**: This study analyzed ten years of domestic research on medical artificial intelligence (AI) from 2015 to 2024 using topic modeling and keyword network analysis. Chronological comparison showed that the research emphasis evolved through three stages—Introduction (2015–2018), Expansion (2019–2022), and Post-ChatGPT (2023–2024)—reflecting the growing incorporation of AI into clinical and service domains. **Methods**: We collected a curated set of 686 papers from the Korea Citation Index (KCI). After preprocessing—stopword removal, synonym unification, and lemmatization—7489 unique terms were extracted for the analysis. **Results**: Topic modeling identified three dominant themes: Diagnostic Imaging and Algorithm Validation, Healthcare Service and System Integration, and Patient-Centered Prediction and Disease Modeling. Keyword network analysis further revealed a structural shift from algorithm-oriented studies to system-level and patient-focused applications. **Conclusions**: These findings indicate that Korean medical AI research is maturing toward a more interpretable, integrated, and human-centered paradigm, underscoring the need for explainable AI (XAI), multidisciplinary collaboration, and governance frameworks for safe and ethical deployment.

## 1. Introduction

Artificial intelligence (AI) has rapidly expanded across the healthcare domain, influencing diagnosis, treatment, patient management, and even health policy [1,2]. In particular, deep learning, natural language processing (NLP), and computer vision technologies have made significant contributions by analyzing vast amounts of medical images and clinical data, thereby improving diagnostic accuracy and offering novel clinical insights [3].

Although international studies on AI in healthcare have grown substantially, many existing reviews remain limited to specific diseases (e.g., cancer and cardiovascular disease) or particular technologies (e.g., medical imaging), restricting a comprehensive understanding of the field [4,5]. In Korea, research on AI-based healthcare has also increased rapidly; however, systematic bibliometric analyses mapping domestic research flows and thematic distributions remain scarce. This gap may hinder the development of evidence-based strategies for future research support and clinical applications at the national level. Therefore, it is necessary to analyze the structure and temporal evolution of healthcare AI research in Korea using a quantitative approach.

In this context, topic modeling, a text mining technique, offers substantial methodological benefits. Topic modeling enables the extraction of latent themes from large-scale academic corpora and provides an overview of structural and temporal trends [6]. Unlike traditional narrative reviews or meta-analyses, topic modeling minimizes researcher subjectivity and systematically organizes extensive text data into interpretable topics [7]. Furthermore, topic modeling facilitates the visualization of inter-topic relationships through network analysis, allowing for a macroscopic understanding of research landscapes [8].

While global studies on medical artificial intelligence (AI) have predominantly focused on algorithmic performance and clinical validation, the contextual determinants shaping AI adoption differ significantly across countries. Korea represents a unique and policy-driven healthcare environment, characterized by a nationally integrated health insurance system, a centralized electronic medical record (EMR) infrastructure, and rapid government-led AI initiatives [9,10,11,12]. These conditions have fostered a distinct ecosystem in which AI technologies are developed and deployed under cohesive national strategies. Unlike the fragmented healthcare systems in many Western countries, Korea’s unified health data governance enables large-scale implementation and consistent policy evaluation.

This unique environment provides an empirical model for understanding how national policy, governance, and academic research interact to accelerate the evolution of AI in medicine. By analyzing Korean research trends over the past decade, this study offers insights into how healthcare AI develops under centralized infrastructure and how this experience may inform global strategies for digital health transformation. Thus, the Korean context is not merely local but serves as a valuable lens through which the international community can examine the balance between innovation, regulation, and ethical implementation of AI in healthcare in general. South Korea’s rapid digital health transformation, driven by its unique demographic shifts and integrated policy landscape, serves as a significant ‘precursor model’ for the international community. This study provides a critical reference point for understanding the global trajectory of medical AI within a highly structured and data-rich healthcare ecosystem.

Accordingly, this study applies topic modeling to the healthcare AI literature indexed in Korean databases to systematically identify the major themes and their temporal evolution, thereby providing a structured overview and practical insights for academic and policy development.

The contributions of this study are as follows. First, we deliver a national-level, longitudinal map of Korean medical AI research (2015–2024) that complements disease- or application-specific reviews. Second, we combine topic modeling and PFNet-based keyword network analysis to characterize both the thematic content and relational structure of keywords over time. Third, we strengthen reproducibility by explicitly documenting topic number selection (coherence C_v profile) and by providing document-level exemplars with topic probabilities. Fourth, we position Korea’s centralized, policy-driven healthcare setting as an informative case for international audiences interested in how research ecosystems translate AI advances into clinical and system integration.

## 2. Research Purpose

This study aims to apply topic modeling to academic research on artificial intelligence (AI) in the medical field published in Korea between 2015 and 2024, allowing us to classify research topics and identify temporal changes. Research in medical AI has diversified along with technological advances, including the adoption of deep learning [1], clinical applications of AI [2,3], and developments in medical imaging and natural language processing (NLP) [4,5]. This study aims, first, to identify the major topics of domestic medical AI research through topic modeling and classify the thematic structures, and second, to analyze the evolution of these topics across three distinct periods: 2015–2018 (Introduction stage), 2019–2022 (Expansion stage, including the COVID-19 pandemic), and 2023–2024 (Post-ChatGPT stage reflecting the emergence of generative AI). Through this dual focus—topic classification and temporal trend analysis—this study provides a comprehensive understanding of the thematic characteristics and developmental trajectory of medical AI research in Korea.

## 3. Materials and Methods

### 3.1. Research Design

This study was designed as a bibliometric analysis to identify thematic structures and temporal changes in Korean medical AI research. This study adopted topic modeling and keyword co-occurrence network analysis as analytical techniques. Topic modeling is a probabilistic model widely used to extract latent topics from large-scale academic corpora and explore research trends quantitatively [6,7,8]. Keyword network analysis is based on the co-occurrence of terms, constructing and visualizing networks to examine inter-topic relationships and structural characteristics of the research field [13,14].

### 3.2. Study Subjects and Data Source

This study targeted peer-reviewed journal articles on medical artificial intelligence (AI) published in Korea between 2015 and 2024. The analytic corpus was constructed from the Korean Citation Index (KCI) platform operated by the National Research Foundation of Korea [15]. The KCI was selected because it provides standardized bibliographic metadata for KCI-indexed journals, enabling a national-level examination of domestic scholarly output. Eligible records were limited to KCI-indexed journal publications within the study period with sufficient text fields for analysis (title and, where available, abstract and keywords). Non-research items such as editorials, corrections, and book reviews were excluded.

### 3.3. Data Collection and Preprocessing

Bibliographic records were retrieved from the KCI platform and restricted to publications from 2015 to 2024. Records were identified by combining multiple AI-related terms as alternative keywords (connected by “OR”) and requiring that at least one medical/healthcare-related term (connected by “OR”) be present simultaneously (combined with “AND”) within the title/abstract/keywords fields (and author keywords when available). The query applied was as follows: (“artificial intelligence” OR “machine learning” OR “deep learning” OR “neural network” OR “natural language processing” OR “NLP” OR “computer vision” OR “large language model” OR “LLM”) AND (“healthcare” OR “medicine” OR “medical” OR “clinical” OR “patient care” OR “diagnosis” OR “diagnostic” OR “treatment” OR “hospital” OR “nursing” OR “nurse”). Retrieved records were exported in CSV format, including publication year, journal, title, abstract, author keywords, and KCI record identifiers/URLs. After removal of duplicate records and exclusion of non-research items, the final analytic corpus comprised 686 articles from an initial pool of 1243 records.

For text mining, titles, abstracts, and keywords were merged into a unified text dataset. Standard preprocessing was conducted to improve term consistency and reduce noise, including stopword removal, synonym merging, and lemmatization. After preprocessing, 7489 unique terms remained for subsequent analyses, including topic modeling and keyword network analysis.

### 3.4. Topic Number Determination

To determine the optimal number of topics, we applied a transparent model selection workflow that combined quantitative coherence evaluation with expert-driven interpretability assessment [6,16]. Candidate LDA models were fitted across *k* = 2–15 under identical estimation conditions, and topic quality for each *k* was evaluated using topic coherence (C_v). Redundancy was assessed by examining whether multiple topics repeatedly produced overlapping top keywords and semantically repetitive themes as *k* increased. Interpretability was assessed using an expert-driven approach. The researcher tested multiple *k* values (*k* = 2–15), ran the model repeatedly, compared the top keywords of each topic across iterations, and selected the final topic number based on expert judgment and interpretability. This assessment prioritized thematic distinctiveness and practical meaning while avoiding excessive fragmentation. The final topic number was determined by jointly considering coherence, redundancy, and interpretability, consistent with methodological practices in applied topic modeling research [16].

### 3.5. Data Analysis

All analyses were conducted using NetMiner 4.5.1 (Cyram Inc., Seongnam, Republic of Korea). Latent Dirichlet Allocation (LDA) was applied to identify latent thematic structures in the corpus [6]. For estimation, all candidate LDA models were fitted using identical hyperparameter settings (α = 0.01, β = 0.01) and Gibbs sampling with 1000 iterations to ensure stable estimation and comparability across topic-number candidates. After selecting the topic number, the final LDA model produced topic–keyword distributions and document–topic probability distributions. To support transparent interpretation, representative documents were identified for each topic by ranking documents according to their topic membership probability, and a document–topic probability table was generated to show the topic probability (“topic prob”) of each representative document. In addition, keyword network analysis was performed to complement the topic modeling results. A co-occurrence network was constructed based on keyword co-occurrence frequencies, and the PathFinder Network Scaling method was applied to reduce network complexity by pruning redundant links [17]. The network was visualized using the Spring map algorithm [18], enabling characterization of thematic structure and temporal evolution.

### 3.6. Ethical Considerations

This study is a bibliometric analysis based on publicly available secondary data. The data used for the analysis were scholarly articles indexed and published in the Korea Citation Index (KCI), all of which are accessible in the public domain. The research did not involve human subjects, human-derived materials, or any personally identifiable information. Therefore, this study was deemed exempt from review by an Institutional Review Board (IRB).

## 4. Results

A total of 686 academic papers on artificial intelligence (AI) in the medical field published in Korea between 2015 and 2024 were analyzed in this study. The findings derived from keyword frequency, topic modeling, and network analysis reveal a rapidly increasing trend in AI-related research and theme diversification over the past decade. This section presents the annual research trends, major keyword frequencies, and structural relationships between research topics.

### 4.1. Annual Research Trends

**Figure 1:**
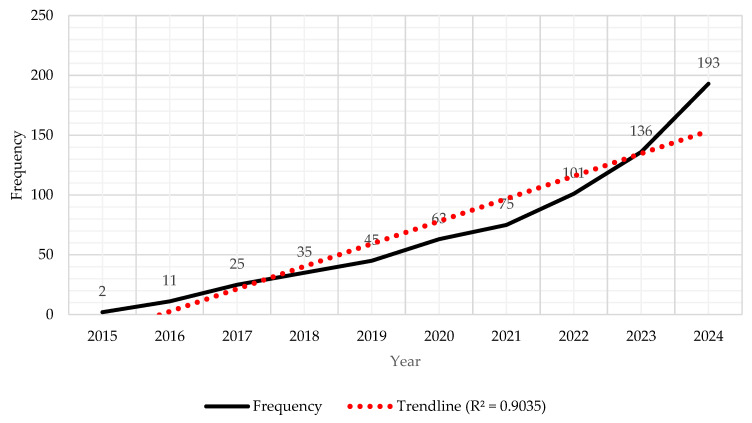
Annual publication trends in medical AI research in Korea (2015–2024). The trendline (R^2^ = 0.9035) indicates a strong linear increase in publication frequency, suggesting consistent and accelerating research activity over the last decade.

Figure 1 illustrates the annual publication trends of medical AI research in Korea from 2015 to 2024. The number of publications increased sharply over time, from only two papers in 2015 to 193 papers in 2024, showing steady and accelerating growth. The fitted linear trendline demonstrated a strong positive correlation (R^2^ = 0.9035), indicating that the temporal increase in publication frequency followed an almost linear growth pattern (Figure 1).

This surge reflects the growing integration of artificial intelligence technologies into the medical and healthcare domains in Korea. The period from 2015 to 2018 represents the introduction stage, when research was limited to conceptual and pilot studies that focused on algorithm development and medical image classification. From 2019 to 2022, corresponding to the expansion stage, the number of publications increased markedly, driven by the proliferation of machine learning applications and the COVID-19 pandemic, which accelerated digital healthcare research. Finally, the Post-ChatGPT stage (2023–2024) exhibits a sharp rise in publications, demonstrating the influence of generative AI and large language models on the medical research landscape.

Overall, this pattern suggests that domestic medical AI research has evolved from exploratory experimentation to systematic, application-driven studies, with growing interdisciplinary collaborations between medicine, computer science, and data engineering.

### 4.2. Top Keywords Analysis

A total of 7489 unique words were extracted from 686 medical AI-related papers published between 2015 and 2024, and their frequencies were examined. Table 1 presents the top 30 keywords that appeared most frequently in the dataset (Table 1).

The word “AI” showed the highest occurrence (*n* = 3497), confirming its centrality and ubiquity in the field. Following “AI,” the terms “diagnosis” (*n* = 652), “healthcare” (*n* = 458), “system” (*n* = 437), and “patient” (*n* = 436) were the most frequent, indicating that diagnostic and healthcare applications are the dominant themes in domestic AI research.

The frequent appearance of terms such as “image,” “algorithm,” “performance,” and “disease” demonstrates the prevalence of studies focusing on deep learning and medical image analysis. The presence of “medicine,” “application,” “development,” and “information” suggests an increasing interest in applying AI technologies to clinical and healthcare settings. In addition, keywords such as “treatment,” “fault diagnosis,” and “classification” reflect the continuous expansion of AI research into therapeutic support and system reliability analysis.

Overall, the keyword frequency results revealed that domestic research on medical AI has primarily concentrated on diagnostic support systems, healthcare management, and algorithmic performance evaluation. This distribution suggests that the Korean research community’s AI studies are highly application-oriented and technically focused, with gradual diversification toward clinical implementation and healthcare innovation.

### 4.3. Keyword Network Analysis

**Figure 2:**
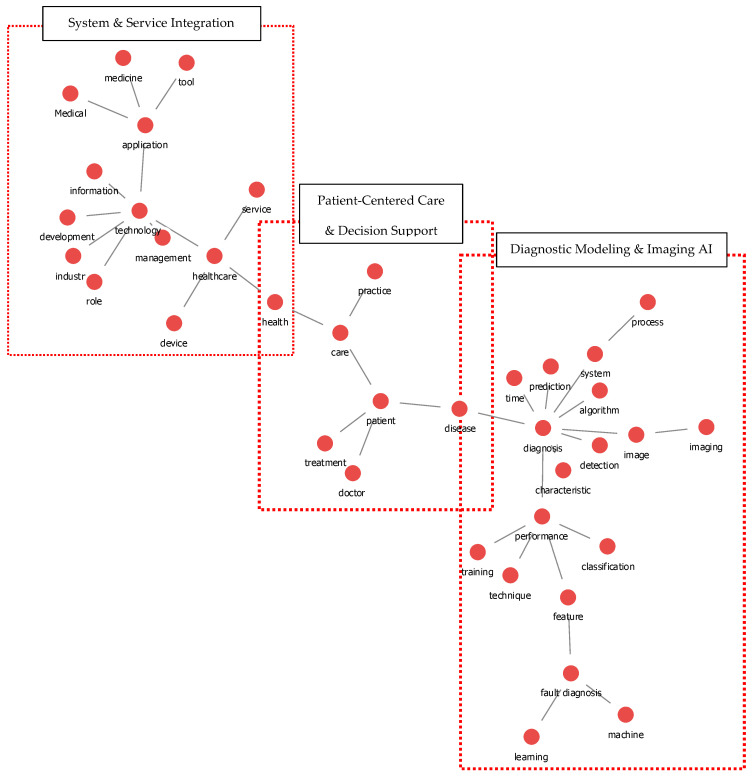
Keyword Co-Occurrence Network of Medical AI Research in Korea (2015–2024). This network map visualizes co-occurrence relationships among frequently appearing keywords extracted from 686 Korean medical AI articles. Cluster–boundary boxes and labels indicate three major thematic groupings: System & Service Integration (**left**), Patient-Centered Care & Decision Support (**center**), and Diagnostic Modeling & Imaging AI (**right**).

A keyword co-occurrence network was constructed to map the intellectual structure of Korean medical AI research based on keywords extracted from 686 articles. A co-occurrence matrix was generated from the frequency with which keywords appeared together within the same documents. To highlight the most meaningful structural relations and prune weak or redundant links, Pathfinder Network Scaling (PFNet) was applied. PFNet retains the shortest (minimum) paths based on semantic distance, allowing the core conceptual structure of the network to be visualized. The resulting PFNet-pruned network is presented in Figure 2.

For clarity, the major clusters in Figure 2 are highlighted with boundary boxes and labeled using the dominant terms in each cluster. Three coherent groupings are visible. The right-side cluster, Diagnostic Modeling & Imaging AI, is organized around the highly connected node diagnosis and closely linked terms such as image, prediction, algorithm, and performance, indicating that diagnosis- and imaging-related modeling remains a central technical axis in Korean medical AI research. The left-side cluster, System & Service Integration, is anchored by healthcare and connected terms such as technology, service, and system, reflecting increasing attention to institutional adoption and service-oriented integration of AI within healthcare environments. The center cluster, Patient-Centered Care & Decision Support, includes terms such as patient, doctor, and care, suggesting a growing emphasis on patient-facing applications and clinical decision-support contexts.

Overall, the cluster-labeled network structure provides a clearer structural reading of the field, where technical development around diagnosis and imaging increasingly coexists with system-level integration and patient-centered clinical applications. Together, these labeled clusters suggest a field structure in which a persistent technical core (diagnostic modeling and imaging) increasingly coexists with institution-level integration and patient-facing clinical applications.

### 4.4. Determination of the Number of Topics

**Figure 3:**
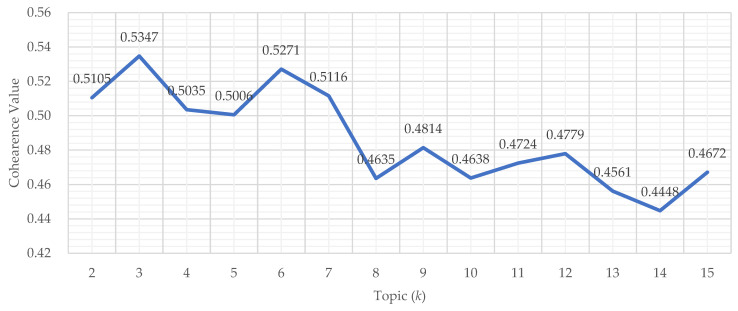
Topic Coherence (C_v) across candidate Topic Numbers (*k* = 2–15). The coherence curve compares candidate LDA models and supports selection of the final topic number.

Topic coherence (C_v) was examined across candidate topic numbers (*k* = 2–15) to support transparent topic number selection (Figure 3). The coherence value reached its maximum at *k* = 3 (C_v = 0.5347), with a secondary local maximum at *k* = 6 (C_v = 0.5271). Within the commonly compared range, the coherence values were 0.5347 (*k* = 3), 0.5035 (*k* = 4), 0.5008 (*k* = 5), and 0.5271 (*k* = 6). For larger topic numbers, coherence generally decreased, suggesting reduced semantic cohesion and increasing topic fragmentation. Considering the coherence profile together with redundancy checks and interpretability review, *k* = 3 was selected as the final topic solution. This coherence profile supports a parsimonious three-topic solution that preserves semantic cohesion while avoiding over-fragmentation.

### 4.5. Topic Modeling Analysis

Topic modeling was conducted to identify latent thematic structures within domestic research on medical artificial intelligence (AI) from 2015 to 2024. As summarized in Table 2, three major topics were derived, each representing a distinct research domain within the field (Table 2).

Topic labels were assigned based on dominant keywords and thematic consistency, and interpretation was further supported by representative documents with high document–topic membership probability (Table 3).

As summarized in Table 2, Topic 1 (Diagnostic Imaging and Algorithm Validation) was characterized by the keywords image, diagnosis, algorithm, fault diagnosis, and performance, indicating a strong emphasis on medical image analysis, diagnostic assistance, and algorithm validation. This theme is exemplified by representative high-probability documents (Table 3), such as “Diagnosis of Colon Polyps Using Artificial Intelligence: Accurate Classification of Diminutive Polyps” (topic prob = 0.9991) and “Data Augmentation Techniques for Deep Learning-Based Medical Image Analyses” (topic prob = 0.9989), illustrating image-based diagnostic applications and methodological work to improve model performance. The representative documents with the highest topic probabilities (Table 3) further illustrate that this topic is anchored in imaging-based diagnostic applications and performance-oriented methodological studies.

Topic 2 (Healthcare Service and System Integration) included the keywords healthcare, technology, medicine, service, and system, reflecting research focused on implementing AI within healthcare delivery and organizational infrastructures. Representative documents (Table 3) such as “Artificial intelligence, Competition law and Healthcare, Pharmaceutical, Medical device markets” (topic prob = 0.9995) and “The Influence of New Service Means on Customer’s Willingness to Buy under the Background of Artificial Intelligence Take the Marketing method of AI medical beauty APP as an example” (topic prob = 0.9994) highlight the expanding scope of medical AI research into system-, market-, and service-oriented domains. Representative high-probability documents (Table 3) indicate that this topic extends beyond clinical tasks to system-, market-, and service-oriented contexts relevant to real-world adoption.

Topic 3 (Patient-Centered Prediction and Disease Modeling) was defined by the keywords diagnosis, patient, disease, performance, and prediction, capturing predictive analytics and patient-centered modeling using health data and machine learning. Representative documents (Table 3), including “Development of a Pressure Injury Machine Learning Prediction Model and Integration into Clinical Practice: A Prediction Model Development and Validation Study” (topic prob = 0.9979) and “The Value of Non-Clinical Applications of Artificial Intelligence in Radiology Should Be Noted” (topic prob = 0.9948), exemplify prediction model development, validation, and clinical integration, as well as applied radiology contexts. The top representative documents (Table 3) demonstrate that this topic emphasizes prediction model development, validation, and integration into clinical practice.

Overall, the joint evidence from keyword-based topic definitions (Table 2) and document-level exemplars (Table 3) indicates that Korean medical AI research has evolved from method- and imaging-driven studies toward broader healthcare system integration and patient-centered predictive applications, reflecting the expanding scope and practical maturation of the field.

### 4.6. Temporal Changes in Topic Proportion

**Figure 4:**
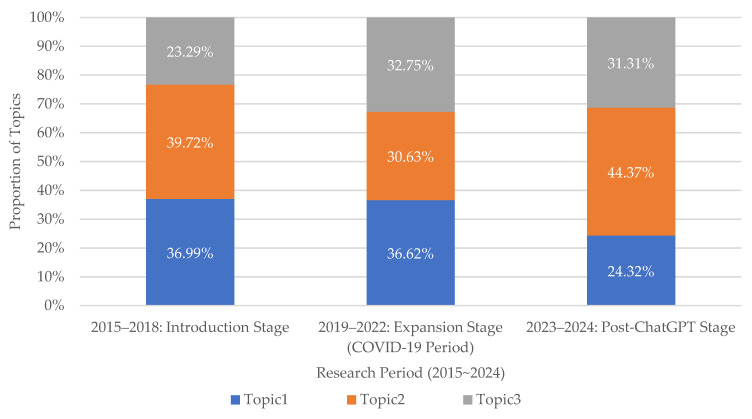
Changes in topic proportion across research stages in medical AI studies (2015–2024). This figure presents the proportion of each topic across three research periods: the Introduction Stage (2015–2018), the Expansion Stage including COVID-19 (2019–2022), and the Post-ChatGPT Stage (2023–2024). The results indicate a gradual transition from technology-oriented studies to patient-centered and service-integration research in recent years.

The temporal distribution of topics was examined by dividing the study period into three stages: 2015–2018 (Introduction Stage), 2019–2022 (Expansion Stage, including the COVID-19 period), and 2023–2024 (Post-ChatGPT Stage). To support transparent interpretation, topic prevalence is reported using both raw counts and proportions across stages (Figure 4).

In the Introduction Stage (2015–2018), the corpus comprised 73 articles, with Topic 1 accounting for 27 (36.99%), Topic 2 for 29 (39.72%), and Topic 3 for 17 (23.29%). In the Expansion Stage (2019–2022), the number of publications increased to 284, and topic shares were distributed as Topic 1 104 (36.62%), Topic 2 87 (30.63%), and Topic 3 93 (32.75%). In the Post-ChatGPT Stage (2023–2024), the corpus further increased to 329 articles, and the distribution shifted to Topic 1 80 (24.32%), Topic 2 146 (44.37%), and Topic 3 103 (31.31%).

Overall, Topic 2 showed the most prominent increase in proportional share in 2023–2024, while Topic 1 decreased relative to earlier stages; Topic 3 remained comparatively stable at approximately one-third of publications in the latter two stages. Because the Post-ChatGPT stage spans only two publication years (2023–2024), the observed proportions should be interpreted as preliminary and may evolve as additional post-2024 publications accumulate.

## 5. Discussion

This study examined ten years of domestic research on medical artificial intelligence (AI) from 2015 to 2024 through topic modeling and keyword network analysis. The results show a continuous evolution of research themes from algorithmic development to system-level integration and patient-centered applications, reflecting methodological advancements and clinical relevance.

During the introduction stage (2015–2018), studies primarily focused on algorithm development and diagnostic imaging, establishing the technical groundwork for medical AI. These studies mirrored early international trends, emphasizing the feasibility and accuracy of deep learning in clinical diagnostics [1,4]. The dominance of performance-oriented research during this period indicates that AI was perceived as a computational aid rather than an embedded component of healthcare.

The expansion stage (2019–2022) marked a shift toward healthcare service optimization, system integration, and patient-centered predictive models. The COVID-19 pandemic accelerated this transition by promoting telemedicine, health data analytics, and AI-assisted diagnostic and surveillance systems [2,3,5]. This stage reflects a conceptual transformation from isolated algorithmic innovation to holistic system implementation, where interoperability and usability within healthcare infrastructure became central research priorities [7,14]. Across stages, we report both raw counts and proportions to contextualize comparisons and reduce the risk of overinterpreting percentage changes observed within limited time windows.

In the Post-ChatGPT stage (2023–2024), the topic distribution shifted, with Topic 2 increasing in share (44.37%, *n* = 146) and Topic 1 decreasing (24.32%, *n* = 80), while Topic 3 remained relatively stable (31.31%, *n* = 103). This pattern may reflect increasing attention to implementation-oriented research, including healthcare service optimization and system-level integration. At the same time, emerging interests in text-based approaches and generative AI have become more visible in the broader medical AI landscape. Because this stage covers only two publication years, these observations should be interpreted as preliminary and may evolve as additional post-2024 publications accumulate.

The cluster-labeled keyword network (Figure 2) provides additional interpretive support for the thematic evolution observed across stages. The Diagnostic Modeling & Imaging AI cluster reflects the field’s continuing technical core around diagnosis- and imaging-related tasks, whereas the System & Service Integration cluster points to an increasing emphasis on institutional adoption, service delivery, and system-level integration. The Patient-Centered Care & Decision Support cluster further suggests a parallel expansion toward clinically contextualized applications that align AI methods with care processes and decision-making needs. Taken together, these clusters depict not a replacement of technical research, but a broadening landscape in which methodological development increasingly coexists with deployment-oriented and patient-centered priorities [19]. This interpretation is consistent with bibliometric observations of international healthcare AI research that report similar differentiation between technical and application-oriented domains [20].

Simultaneously, recent scholarship has highlighted explainability and interpretability as critical challenges for sustainable AI integration in medicine. As Markus et al. [21] and Sadeghi et al. [22] argue, model transparency is essential for building clinician trust, ensuring safety, and supporting regulatory compliance. Explainable AI (XAI) methods, such as SHAP or LIME, are increasingly adopted to bridge the gap between model performance and clinical interpretability [23]. Without such transparency, AI systems risk remaining “black boxes,” which could hinder their adoption, accountability, and long-term credibility within clinical workflows.

Beyond domestic implications, these findings contribute to the global discourse on policy-driven AI ecosystems. Korea’s national-level data standardization, extensive electronic health record adoption, and coordinated AI policies, such as the Digital New Deal and AI-Based Precision Medicine Projects, demonstrate how centralized strategies can accelerate academic and clinical innovation [10,11,12,19]. The country’s cohesive digital infrastructure not only facilitates rapid deployment but also enables the systematic evaluation of the ethical, legal, and technical aspects of AI in medicine. Furthermore, these findings are broadly consistent with international discussions describing a diversification of healthcare AI research beyond imaging-focused diagnostics toward text-based data and foundation model applications in clinical documentation, administration, and education [24]. The increasing emphasis on system integration and patient-centered applications observed in Korea may reflect a maturation trajectory that has also been noted in global bibliometric and review studies, which highlight the growing importance of implementation contexts and interdisciplinary collaboration in medical AI [25]. Recent reviews in nursing informatics similarly suggest that the field is moving beyond basic digitalization toward more integrated, AI-supported practice and administrative workflows [26]. Taken together, the Korean case provides an empirical reference for how research themes may evolve within a policy-driven and digitally mature healthcare environment, while underscoring the need for continued cross-national comparison.

However, this centralization also introduces emerging challenges related to transparency, data ownership and accountability. As other nations seek to emulate similar frameworks, Korea’s experience provides a practical reference model for balancing innovation and governance issues. From a methodological standpoint, the topic modeling and keyword network analysis employed in this study offer a replicable framework for cross-national comparison. Expanding such analyses to other healthcare systems could help establish a global model of AI research evolution, illustrating how institutional and policy structures shape the trajectory of technological advancement in medical contexts.

In summary, this study reveals that domestic medical AI research has transitioned from a technology-centric phase to a system- and service-oriented phase that prioritizes patient outcomes, interpretability, and integration. To sustain this momentum, future research should emphasize the following:

(1) Multidisciplinary collaboration among clinicians, data scientists, and policymakers; (2) explainable and trustworthy AI frameworks to enhance usability and ethics; and (3) longitudinal monitoring of emerging topics, such as federated learning, multimodal AI, and generative models for medical reasoning.

These directions will ensure that the next generation of medical AI systems advances not only in terms of capability but also in terms of transparency, fairness, and clinical relevance.

## 6. Limitations

This study sought to elucidate the medical AI research landscape in South Korea, a unique context characterized by centralized data governance and national-level AI initiatives. The goal was to present international audiences with insights into how technology adoption and policy influenced the evolution of research themes.

Nevertheless, the findings of this study should be interpreted considering several limitations.

First, our analysis was confined to publications indexed in the Korea Citation Index (KCI) to identify domestic research trends. While the KCI is a representative database for the national academic ecosystem, papers by Korean researchers published in major international journals (e.g., Scopus and Web of Science) were excluded. This exclusion may result in thematic differences or time lags in the technological trends between domestic and international research. Therefore, caution should be exercised when generalizing these findings to the entire body of Korean medical AI research.

Second, the topic modeling analysis carries methodological limitations. The final number of topics (three) was determined based largely on interpretability, that is, the researcher’s judgment regarding the distinctiveness and practical meaning of each topic, rather than a mechanical reliance on statistical metrics. This approach aligns with prior studies [8] that prioritize the discovery of “meaningful themes” over pure statistical optimization. However, we acknowledge that this process involves researcher subjectivity, and the resulting topic structure may vary if different interpretive criteria are applied.

In addition, the absence of an international comparative analysis should be interpreted as a defined next step rather than only a limitation. While restricting the corpus to KCI-indexed journals enables a focused examination of domestically indexed scholarship that is closely connected to Korea’s healthcare system and policy context, Korean-authored publications in international journals were not included, which may influence the observed topic distribution. Internationally published studies may more frequently emphasize large-scale external validation, imaging-intensive benchmarking, and multi-institutional clinical evaluation, potentially increasing the relative weight of technically oriented themes (e.g., diagnostic modeling and imaging AI). In contrast, domestically indexed journals may more prominently capture system-, service-, and policy-facing discussions and locally contextualized clinical applications. Future work incorporating international databases (e.g., Web of Science, Scopus, or PubMed) will be valuable to assess the robustness of topic prevalence and to conduct comparative analyses between domestic and international publication ecosystems.

## 7. Conclusions

This study analyzed domestic research trends in medical artificial intelligence (AI) from 2015 to 2024 using topic modeling and keyword network analysis. By classifying ten years of publications into three chronological stages—Introduction (2015–2018), Expansion (2019–2022), and Post-ChatGPT (2023–2024)—the results revealed a clear evolution of research priorities from algorithm-centered development to system integration and patient-centered applications.

Early studies focused on algorithmic validation and diagnostic imaging, reflecting Korea’s initial technological foundation in medical AI. Over time, research has expanded toward healthcare service optimization, predictive modeling, and clinical decision support, culminating in recent explorations of large language models and generative AI for healthcare communication and knowledge retrieval purposes. Keyword network analysis further confirmed this trajectory, showing a structural shift from technical to service-oriented themes in the literature.

These findings demonstrate that domestic medical AI research is transitioning into a mature phase, emphasizing transparency, interpretability, and clinical integration. Future studies should strengthen multidisciplinary collaboration, apply explainable AI (XAI) methods to ensure accountability and trust, and establish governance frameworks for safe and equitable AI deployment in healthcare. By doing so, Korean medical AI research is expected to contribute not only to technological advancement but also to the creation of a sustainable and human-centered healthcare ecosystem. Ultimately, the Korean case study serves as a valuable empirical basis for establishing global standards in the implementation of medical AI. These results underscore that the successful global deployment of healthcare AI depends on developing frameworks that are both locally relevant and internationally compatible, ensuring a sustainable and equitable digital transformation across diverse healthcare environments.

## Figures and Tables

**Table 1:** Top 30 keywords in medical AI research (2015–2024). Keywords were extracted from **686** KCI-indexed articles published between 2015 and 2024 and ranked by frequency.

Rank	Word	Frequency (*n*)	Rank	Word	Frequency (*n*)
1	AI	3497	16	treatment	226
2	diagnosis	652	17	fault diagnosis	224
3	healthcare	458	18	health	223
4	system	437	19	service	213
5	patient	436	20	classification	211
6	image	435	21	prediction	179
7	technology	405	22	detection	175
8	algorithm	390	23	care	170
9	performance	371	24	device	165
10	disease	315	25	fault	163
11	medicine	305	26	process	162
12	application	275	27	technique	158
13	development	266	28	doctor	148
14	information	262	29	signal	135
15	feature	230	30	time	129

**Table 2:** Topic classification and representative keywords in medical AI studies (2015–2024). This table summarizes the three extracted topics derived from Latent Dirichlet Allocation (LDA) modeling. Each topic is labeled according to its dominant terms and thematic coherence: Topic 1—Diagnostic Imaging and Algorithm Validation, Topic 2—Healthcare Service and System Integration, and Topic 3—Patient-Centered Prediction and Disease Modeling.

Topic	1st Keyword	2nd Keyword	3rd Keyword	4th Keyword	5th Keyword
Topic 1: Diagnostic Imaging and Algorithm Validation	image	diagnosis	algorithm	fault diagnosis	performance
Topic 2: Healthcare Service and System Integration	healthcare	technology	medicine	service	system
Topic 3: Patient-Centered Prediction and Disease Modeling	diagnosis	patient	disease	performance	prediction

**Table 3:** Representative documents per topic (ranked by topic probability, “topic prob”). Representative documents were selected based on the highest document–topic membership probability for each topic.

Topic	Representative Documents	Topic Prob.
Topic 1	Diagnosis of Colon Polyps Using Artificial Intelligence: Accurate Classification of Diminutive Polyps	0.9991
	Data Augmentation Techniques for Deep Learning-Based Medical Image Analyses	0.9989
Topic 2	Artificial intelligence, Competition law and Healthcare, Pharmaceutical, Medical device markets	0.9995
	The Influence of New Service Means on Customer’s Willingness to Buy under the Background of Artificial Intelligence Take the Marketing method of AI medical beauty APP as an example	0.9994
Topic 3	Development of a Pressure Injury Machine Learning Prediction Model and Integration into Clinical Practice: A Prediction Model Development and Validation Study	0.9979
	The Value of Non-Clinical Applications of Artificial Intelligence in Radiology Should Be Noted	0.9948

## Data Availability

The data used for the analysis were scholarly articles indexed and published in the Korea Citation Index (KCI), which are accessible in the public domain at https://www.kci.go.kr (accessed on 15 January 2026).
